# ZZ/ZW Sex Chromosomes in the Madagascar Girdled Lizard, *Zonosaurus madagascariensis* (Squamata: Gerrhosauridae)

**DOI:** 10.3390/genes14010099

**Published:** 2022-12-29

**Authors:** Alexander Kostmann, Lukáš Kratochvíl, Michail Rovatsos

**Affiliations:** Department of Ecology, Faculty of Science, Charles University, 12844 Prague, Czech Republic

**Keywords:** C-banding, CGH, cytogenetics, FISH, karyotype, rDNA loci, sex chromosomes, sex determination, telomeres, ZZ/ZW

## Abstract

Scincoidea, the reptilian clade that includes girdled lizards, night lizards, plated lizards and skinks, are considered as a lineage with diversity in sex-determining systems. Recently, the hypothesis on the variability in sex determination in skinks and even more the absence of sex chromosomes in some of them has been rivalling. Homologous, evolutionary stable XX/XY sex chromosomes were documented to be widespread across skinks. However, sex determination in the other scincoidean families is highly understudied. ZZ/ZW sex chromosomes have been identified only in night lizards and a single species of plated lizards. It seems that although there are different sex chromosome systems among scincoidean lineages, they share one common trait: they are mostly poorly differentiated and often undetectable by cytogenetic methods. Here, we report one of the exceptions, demonstrating for the first time ZZ/ZW sex chromosomes in the plated lizard *Zonosaurus madagascariensis*. Its sex chromosomes are morphologically similar, but the W is clearly detectable by comparative genomic hybridization (CGH), suggesting that the Z and W chromosomes highly differ in sequence content. Our findings confirm the presence of female heterogamety in plated lizards and provides novel insights to expand our understanding of sex chromosome evolution in scincoidean lizards.

## 1. Introduction

Sex determination in amniotes ranges from environmental sex determination to genotypic sex determination with highly differentiated sex chromosomes [1,2,3,4]. Sex chromosomes probably evolved many times within amniotes, although the reconstruction of the ancestral sex determination and the evolutionary transitions among sex determining modes are still a matter of debate [5,6]. Despite the constant progress, our understanding of the evolution of sex chromosomes is complicated by a lack of data in many extant lineages [4,5,6]. An emerging pattern is that some lineages such as birds, caenophidian snakes, iguanas, lacertids, mammals and monitors have stable, differentiated sex chromosomes for a long evolutionary time [7,8,9,10,11,12]. On the other hand, some reptilian lineages exhibit variability in sex determination, which can be attributed to their old age and quite ancient transitions in sex determination [13] or to relatively frequent turnovers of sex chromosomes [14,15]. Recently, the attention has been directed to one of the last highly diversified clades among squamate reptiles which has been understudied in this regard: the scincoidean lizards.

Scincoidean lizards are a species-rich and morphologically diversified group, comprising girdled lizards (Cordylidae), night lizards (Xantusiidae), plated lizards (Gerrhosauridae) and skinks (Scincidae). Girdled lizards possess approximately 70 species, distributed in sub-Saharan Africa [16]. Night lizard lizards are a small family with 37 species distributed in the North and Central America and adjacent islands [16]. Plated lizards are classified into 37 species divided into two subfamilies: Gerrhosaurinae, a group of species distributed widely across sub-Saharan Africa, and Zonosaurinae, a group of lizards restricted to Madagascar and nearby islands [16,17]. Skinks show a nearly cosmopolitan distribution, and with around 1700 species cover around 15% of the total reptile species diversity [16].

Although many scincoidean lizards have been cytogenetically studied [18,19,20,21,22,23,24,25,26,27,28,29], sex chromosomes were identified in only approx. 20 species [28,29]. XX/XY sex chromosomes were identified by cytogenetic methods in a few species of skinks based mainly on differences between X and Y in morphology and distribution of repetitive elements [26,27,30]. On the other hand, heteromorphic ZZ/ZW sex chromosomes were identified in *Scincella melanosticta*, the only species of skinks with known female heterogamety [31]. Whole-genome sequencing and subsequent genomic coverage analysis led to the identification of X-specific gene content in two species of skinks: *Scincus scincus* and *Eulamprus heatwolei* [27,32]. A subsequent qPCR-based analysis revealed homologous XX/XY sex chromosomes in 13 representative species of skinks, covering most of the major phylogenetic lineages of this group [27]. These poorly differentiated sex chromosomes in skinks have remained evolutionary stable for at least 85 million years [27]. In night lizards, ZZ/ZW sex chromosomes were identified in *Xantusia henshawi* based on single nucleotide polymorphisms (SNPs) derived from restriction site-associated DNA sequencing (RADseq) [33], and female heterogamety was also predicted in *Lepidophyma smithii*, because females can produce by facultative parthenogenesis offspring of both sexes [34]. In plated lizards, we detected ZZ/ZW sex chromosomes in *Tracheloptychus petersi* by cytogenetic methods due to differential accumulation of rDNA loci between the Z and the W chromosomes [29]. Notably, sex chromosomes have not been reported in any girdled lizard yet.

In this study, we examined the Madagascar girdled lizard, *Zonosaurus madagascariensis* (Gray, 1831), with both conventional (karyogram reconstruction, C-banding) and molecular (fluorescence in situ hybridization—FISH, comparative genome hybridization—CGH) cytogenetic methods, with emphasis to reveal sex chromosomes.

## 2. Materials and Methods

### 2.1. Samples and Species Verification

We collected blood samples from five adult individuals (two males and three females) of *Z. madagascariensis*. Their sex was determined by external morphology (i.e., prominent and/or active femoral pores in males) and by everting of hemipenes (i.e., the male-specific reproductive organs) by palpation in males. The correct sex identification was later confirmed by gonad inspection in four individuals. Total DNA was extracted from blood samples using the DNeasy Blood and Tissue Kit (Qiagen, Hilden, Germany).

### 2.2. Chromosome Preparation and Staining

We prepared mitotic chromosome spreads from whole-blood cell cultures, following the protocol previously described in Kostmann et al. [26]. Metaphases were stained with 5% Giemsa to visualize chromosome morphology and to prepare karyograms, and C-banding to visualize the heterochromatic regions, following the protocol of Sumner [35] with slight modifications [26].

### 2.3. Fluorescence In Situ Hybridization with Probes for rDNA Loci and Telomeric Repeats

The probe for rDNA loci was prepared from a plasmid (pDmr.a 51#1) with an 11.5-kb insert encoding the 18S and 28S ribosomal units of *Drosophila melanogaster* [36] labeled by nick-translation (Abbott Laboratories, Lake Bluff, IL, USA) with dUTP-biotin (Roche, Basel, Switzerland). The probe for telomeric repeats was prepared by PCR, according to the protocol previously published in Rovatsos et al. [37]. The protocol for in situ hybridization is presented in detail in Kostmann et al. [26].

### 2.4. Comparative Genomic Hybridization

Total DNA extraction from males was labeled with dUTP-biotin (Roche, Basel, Switzerland), while from females with dUTP-digoxigenin (Roche, Basel, Switzerland), using a commercial kit for nick-translation (Abbott Laboratories, Lake Bluff, IL, USA). Labelled DNA from one male and one female was mixed in equal concentration and purified with ethanol/cold precipitation. Our detailed protocol for probe preparation and in situ hybridization conditions was previously published in Kostmann et al. [26]. CGH was applied to chromosome spreads from both male and female individuals to identify sex-specific genomic regions.

### 2.5. Microscopy and Image Analyses

We captured at least 10 metaphases for each studied specimen and applied method, by either a Zeiss Axio Imager Z2 equipped with automatic Metafer-Msearch scanning platform equipped with a CoolCube 1 b/w digital camera (MetaSystems, Altlussheim, Germany) or a Provis AX70 fluorescence microscope equipped with a DP30BW digital camera (Olympus, Tokyo, Japan).

## 3. Results

**Figure 1:**
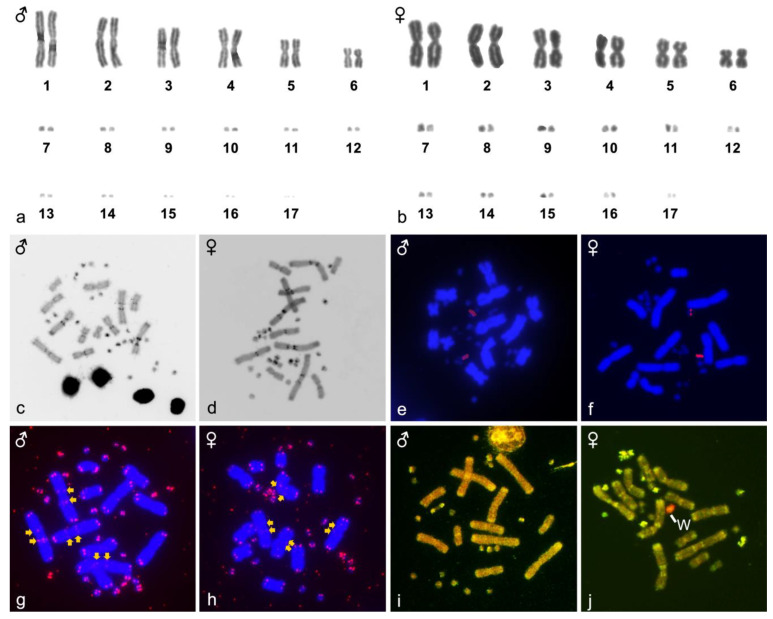
Giemsa-stained karyograms (**a**,**b**), C-banding (**c**,**d**), rDNA loci (**e**,**f**), telomeric motifs (**g**,**h**) and comparative genomic hybridization (**i**,**j**) in metaphases of a male and a female *Z. madagascariensis*. The W chromosome is indicated in CGH. ITRs are indicated by yellow arrows.

All five studied individuals of *Z. madagascariensis* have identical karyotype with 2n = 34 chromosomes; 12 metacentric macrochromosomes and 22 microchromosomes (Figure 1a,b). Heterochromatin is located in centromeres and prominent blocks were detected in the pericentromeric regions of the first five largest pairs. In addition, at least four pairs of microchromosomes show strong accumulation of heterochromatin (Figure 1c,d). Fluorescence in situ hybridization with a probe for the rDNA loci detected signals in a single pair of microchromosomes in both sexes (Figure 1e,f). The telomeric repeats were detected in the expected terminal position of all chromosomes, but additionally, interstitial telomeric repeats (ITRs) were observed in the chromosome pairs 1 and 2 (Figure 1g,h). The comparative genome hybridization revealed extensive female-specific signal covering a single, large-sized microchromosome (Figure 1i,j).

## 4. Discussion

All five studied individuals of *Z. madagascariensis* show identical karyotypes, in terms of diploid number and chromosome morphology, which is in accordance with the karyotype previously published for this species by Odierna et al. [24]. The karyotype of 2n = 34 chromosomes with 12 bi-armed macrochromosomes and 22 microchromosomes is common across the majority of girdled lizards and plated lizards, and probably corresponds to the ancestral karyotype for this group [24]. The rare deviations from this putative ancestral karyotype are usually species-specific apomorphies, involving mainly centromeric fission of macrochromosomes (e.g., in *Cordylus cataphractus* and *Cordylus giganteus*) and the formation of additional pairs of microchromosomes (e.g., in *Gerrhosaurus flavigularis*) [20,21,24]. Nevertheless, similarities in chromosome morphology in Giemsa-stained karyograms is not a decisive proof of homology between genomic regions, as inter- and intra- chromosomal rearrangements may occur without necessary changing the chromosome morphology. Notable cases of chromosomal rearrangements that may not change chromosome morphology are for example pericentromeric inversions [38] and whole-arm reciprocal translocations (WARTs) [39]. 

Heterochromatin is typically expected in centromeres, but extensive heterochromatic blocks in pericentromeric regions and microchromosomes are common in reptiles, as previously reported in several species of chameleons [40], beaded lizards, monitors [41], snakes [42] and the plated lizard *T. petersi* [29]. The evolutionary history and function of heterochromatin accumulation in pericentromeric regions and microchromosomes is unclear. Extensive heterochromatinization is a common mechanism to silence transposons [43,44] and often occurs in genomic regions enriched in satellite motifs [45]. Therefore, we can speculate that the heterochromatin blocks of *Z. madagascariensis* should be enriched in repetitive elements. Furthermore, non-centromeric heterochromatic regions are often enriched with telomeric-like sequences [46,47], which is also the case in chromosome pairs 1 and 2 of *Z. madagascariensis* (Figure 1). Telomeric repeats in inner chromosome positions are often interpreted as remnants of chromosome rearrangements, such as inversions and chromosome fusions. Chromosome rearrangements can directly transfer terminal telomeric sequences to inner positions or can induce indirectly *de novo* synthesis of telomeric motifs during the process of DNA repair [48,49,50,51].

The rDNA loci tend to form long tandem repeats in the genome, and therefore, they are easily detectable by in situ hybridization. The majority of the reptilian species show hybridization signals in a single pair of chromosomes, but occasionally in two pairs and extremely rarely in more [24,42,52,53,54,55]. rDNA loci may accumulate on sex chromosomes, where the Y/W differentiation process may lead to a sex-specific pattern, such as in chelid turtles [56], trionychid turtles [57,58] and skinks [26,27]. Therefore, the comparative examination of the topology of rDNA loci between sexes can reveal sex chromosomes. Nevertheless, no sex-specific pattern of rDNA loci was detected in *Z. madagascariensis*.

Comparative genomic hybridization has been widely used to identify sex chromosomes in a plethora of animal taxa, ranging from insects to mammals, including both XX/XY and ZZ/ZW systems [59,60]. This method was applied to detect sex chromosomes in many species of reptiles, including geckos [61,62,63], caenophidian snakes [64,65], chameleons of the genus *Furcifer* [66], chelid turtles [56], beaded lizards [41], iguanas [67], lacertid lizards [68], monitors [41,69] and the dragon lizard *Pogona vitticeps* [70]. In the current study, we detected female-specific signal in a single microchromosome by CGH, which should correspond to the W chromosome (Figure 1). The CGH signal on the W is quite intense, and therefore we assume that the Z and W chromosomes should differ in sequence content. Such differentiated W chromosomes tend also to be heterochromatic, but after re-examining our C-banded metaphases, we cannot distinguish the W chromosome due to its small size and similar pattern in multiple microchromosomes (Figure 1). Notably, another studied plated lizard, *T. petersi*, has also ZZ/ZW sex determination [29]. In contrast to *Z. madagascariensis*, CGH did not reveal the sex chromosomes in *T. petersi*, but instead the W chromosome in *T. petersi* is missing the accumulation of rDNA loci, which is detected at the Z chromosome [29]. In both these species of plated lizards, the sex chromosomes seem to be a pair of microchromosomes, but with the current state of knowledge, we cannot conclude if they are homologous. If yes, the sex chromosomes could be at least 34 million years old, which is an estimated age of the split between the genera *Zonosaurus* and *Tracheloptychus* [71].

Such variability in the pattern of CGH and rDNA loci between species with homologous sex determination has been previously documented in other species as well. For example, CGH did not detect sex chromosomes in five species of skinks (*Eutropis multifasciata*, *Scincopus fasciatus*, *S. scincus*, *Tropidophorus baconi* and *Tiliqua gigas*), despite that they all share homologous XX/XY sex chromosomes stable for at least 85 million years [26,27]. A plausible explanation is that the Y chromosome in skinks has a low level of differentiation in comparison to the X chromosome, and therefore, such small sex-specific differences are below the detection limit of CGH. Similarly, despite all iguanas of the genus *Oplurus* share homologous XX/XY sex chromosomes, the Y chromosome is detectable by CGH in *Oplurus cuvieri*, but not in *Oplurus fierinensis* [67]. In addition, the topology and sexual differences in rDNA loci might vary even between closely related species. For example, rDNA loci accumulate only on the X chromosome and are missing on the Y in *S. scincus*, but appear in a single pair of chromosomes without any notable sex-specific differences in other species of skinks [26,27].

In summary, with the exception of ZZ/ZW in *S. melanosticta* [31] and the effect of environmental factors on sex determination in few species [28,72], all other tested species of skinks seem to have XX/XY sex chromosomes, evolved from genomic region homologous to chicken chromosome 1 [27]. In contrast, up to now, only ZZ/ZW sex chromosomes have been reported in the sister lineage to skinks, i.e., the clade including night lizards and the sister families Cordylidae and Gerrhosauridae, but the knowledge in them is very limited. The ZZ/ZW sex chromosomes in *X. henshawi* evolved from genomic regions homologous to parts of chicken chromosomes 7, 12 and 18 [33]. In addition, ZZ/ZW is expected in another night lizard, *L. smithii*, but the gene content and homology of sex chromosomes remains unknown [34]. To the best of our knowledge, sex chromosomes have not been identified yet in girdled lizards. ZZ/ZW sex chromosomes have been also reported in two plated lizard species ([29], current study), but the homology of the sex chromosomes between these two species and to night lizards remains unknown. The emerging pattern of sex determination in Scincoidea allows to speculate that GSD was probably their ancestral system, but in order to further reconstruct the possible scenaria of the evolution of sex chromosomes in this group, additional cytogenetic (e.g., comparative chromosome painting) and genomic analysis (e.g., whole genome sequencing and comparative genome coverage analysis and/or analysis of SNPs in data from restriction site-associated DNA sequencing) are needed to identify the sex chromosome gene content in girdled lizards, plated lizards and additional species of night lizards.

## Data Availability

Not applicable.
